# Overcoming antibiotic resistance through efficient and scalable total synthesis

**DOI:** 10.1093/nsr/nwac029

**Published:** 2022-02-23

**Authors:** Yang Jiao, Tuoping Luo

**Affiliations:** Key Laboratory of Bioorganic Chemistry and Molecular Engineering, Ministry of Education and Beijing National Laboratory for Molecular Science, College of Chemistry and Molecular Engineering, Peking University, China; Key Laboratory of Bioorganic Chemistry and Molecular Engineering, Ministry of Education and Beijing National Laboratory for Molecular Science, College of Chemistry and Molecular Engineering, Peking University, China; Peking-Tsinghua Center for Life Sciences, Academy for Advanced Interdisciplinary Studies, Peking University, China; Institute of Molecular Physiology, Shenzhen Bay Laboratory, China

**Figure 1.**
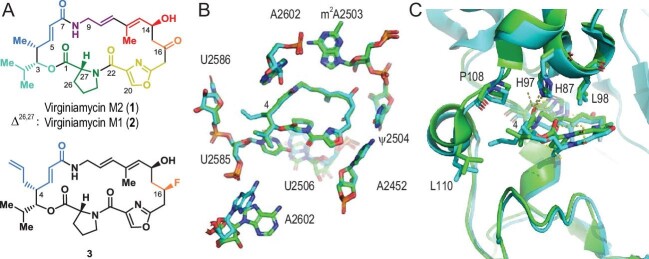
(A) Structures of virginiamycins M2 (**1**), M1 (**2**) and the synthetic analog (**3**) with reduced VatA susceptibility. Fragments are colored differently. (B) The cryo-EM structures of **1** (green) and **3** (cyan) bond to the 50S subunit of the *E. coli* ribosome (PDB codes 6PCQ and 6PC6, respectively). (C) X-ray crystal structures of **2** (green) and **3** (cyan) bound to VatA (PDB codes 4HUS and 6 × 3C, respectively).

Bacterial ribosome has served as an important target for antibiotics that are essential to public health, such as erythromycins, tetracyclines, aminoglycosides, pleuromutilin and fusidic acid, all with natural origin [1]. The structural complexity of these natural products has posed significant challenges to total synthesis, especially in an efficient and scalable manner, hence their semi-synthetic analogs dominating the clinical setting. The scenario has undergone a paradigm shift over recent years, starting with the landmark work on tetracyclines reported by Myers’ group, which led to novel analogs via *de novo* total synthesis [2]. However, for group A streptogramin antibiotics, such as virginiamycin M2 (**1**, Fig. 1A) featuring a 23-membered macrocyclic ring, a conjugated dienyl and an oxazole motif, even though corresponding total synthesis emerged over two decades ago [3], these fully synthetic routes have not been applied in medicinal chemistry optimization. Group A bind to the peptidyl transferase center in synergy with the group B streptogramins to manifest the bactericidal effect, and bacteria tend to develop high-level resistance to the A component compared to the B component, a specific mechanism of which is deactivation by resistance enzyme virginiamycin acetyltransferases (Vats). Consequently, overcoming bacterial resistance is a goal that has been intensely pursued, and requires not only an understanding of the different structural requirements with regard to the interaction between bacterial ribosome and Vats, but also practical synthetic approaches to modifying the scaffold at will.

Recently, Seiple and co-workers have combined chemical synthesis, high-resolution structural determination of target-ligand complexes, and *in vitro* and *in vivo* biological evaluations to address this problem [4]. They first solved the cryo-electron microscopy (cryo-EM) structure of **1** bound to the *Escherichia coli* 50S ribosome (Fig. 1B), suggesting that C3 isopropyl (projecting towards the tRNA P-site), C4 methyl and C6 hydrogen on **1** had less interaction with ribosomes, but were more important for VatA binding (particularly, C4 stays close to L110 residue when **1** binds to VatA). Therefore, modifications of these positions were expected to reduce bacterial resistance without affecting the antibiotic efficacy.

Their original synthetic route to **1**, by assembling seven building blocks [3], was streamlined to afford over 60 new group A streptogramin analogues: (i) the C3 and C4 stereogenic centers were secured through a Mukaiyama Aldol reaction under the asymmetric catalysis of a proline-based oxazaborolidine; (ii) a terminal hydroxyl group could be introduced at the C3 substituent by switching isobutylaldehyde into 3-hydroxy-2-methyl-propanal, which could be further derivatized via isocyanate or S_N_2 reactions; (iii) the methyl group at C4 could be replaced with an allyl group; (iv) the C14 stereogenic center bearing a hydroxyl group was constructed using an Evans Aldol reaction, followed by the introduction of the C16 carbonyl group that could subsequently be reduced and fluoridated to provide analogues of flopristin, a clinical candidate; (v) amide and ester bond couplings were followed by a Stille macrocyclization reaction to complete the synthesis.

Subsequent evaluation of antimicrobial spectra and *in vitro* ribosomal translation inhibitory activities advanced the knowledge of structure–activity relationships, and prioritized compounds for follow-up studies, including the combination with the group B streptogramin, evaluation in multidrug-resistant clinical isolates and an *in vivo* mouse model of a *Staphylococcus aureus* infection. The most promising analogues, such as compound **3**, were further investigated in terms of the mechanism of action and resistance. The cryo-EM structure of **3** binding to *E. coli* ribosome confirmed the extension and the potential interactions of C4-allyl towards the streptogramin B binding site (Fig. 1B). The X-ray co-crystal structures of **2** and **3** binding to VatA revealed that the C4 allyl group of **3** had a steric clash with L110 residue and adopted a more strained conformation than that of the ribosome-bound one (Fig. 1C), rationalizing the reduced rate of C14 acetylation at **3** by VatA.

In conclusion, the report by Seiple and co-workers reinforces the idea that efficient exploration into the chemical space of complex natural products, once considered a daunting task, becomes feasible with the progress of synthetic methodology and strategy, providing a solid foundation with which to take advantage of these chemotypes co-evolving with life. In order to meet the pressing needs of human health, such as the urgent call for new antibiotics to combat bacterial resistance, the enabling capability of total synthesis, in combination with state-of-the-art technologies such as cryo-EM and artificial intelligence [5], could serve as an integrated approach to facilitate the identification of new candidates for drug discovery process.

***Conflict of interest statement*.** None declared.
